# Negative Impact of Hypoxia on Tryptophan 2,3-Dioxygenase Function

**DOI:** 10.1155/2016/1638916

**Published:** 2016-08-02

**Authors:** Frank Elbers, Claudia Woite, Valentina Antoni, Sara Stein, Hiroshi Funakoshi, Toshikazu Nakamura, Gereon Schares, Walter Däubener, Silvia K. Eller

**Affiliations:** ^1^Institute of Medical Microbiology and Hospital Hygiene, Heinrich-Heine-University, 40225 Düsseldorf, Germany; ^2^Center for Advanced Research and Education, Asahikawa Medical University, Asahikawa 078-8510, Japan; ^3^Neurogen Inc., 1-1-52-201 Nakahozumi, Ibaraki 567-0034, Japan; ^4^Friedrich-Loeffler-Institut, Federal Research Institute for Animal Health, Institute of Epidemiology, 17493 Greifswald-Insel Riems, Germany

## Abstract

Tryptophan is an essential amino acid for hosts and pathogens. The liver enzyme tryptophan 2,3-dioxygenase (TDO) provokes, by its ability to degrade tryptophan to N-formylkynurenine, the precursor of the immune-relevant kynurenines, direct and indirect antimicrobial and immunoregulatory states. Up to now these TDO-mediated broad-spectrum effector functions have never been observed under hypoxia* in vitro*, although physiologic oxygen concentrations in liver tissue are low, especially in case of infection. Here we analysed recombinant expressed human TDO and* ex vivo* murine TDO functions under different oxygen conditions and show that TDO-induced restrictions of clinically relevant pathogens (bacteria, parasites) and of T cell proliferation are abrogated under hypoxic conditions. We pinpointed the loss of TDO efficiency to the reduction of TDO activity, since cell survival and TDO protein levels were unaffected. In conclusion, the potent antimicrobial as well as immunoregulatory effects of TDO were substantially impaired under hypoxic conditions that pathophysiologically occur* in vivo*. This might be detrimental for the appropriate host immune response towards relevant pathogens.

## 1. Introduction

There is great interest in understanding the composition of the tissue microenvironment and its consequence for immune responses. One of the most important microenvironmental factors is the oxygenation status of tissues, since oxygen affects a plethora of cellular processes including innate and adaptive cellular immune defence mechanisms. Cells in different tissues are exposed to a wide range of oxygen concentrations, and local oxygen concentrations between 1 and 12% O_2_ are physiologic [1]. In particular the liver has a unique anatomical structure that creates an oxygen gradient between 8% O_2_ and 4% O_2_ within the liver compartments and the pO_2_ is even more reduced during infection to ≤1% O_2_ [2, 3].

Human tryptophan 2,3-dioxygenase (TDO) is a liver enzyme with a well-described function in tryptophan homeostasis and crucial immunoregulatory features. The latter was shown* in vitro* [4] and* in vivo*. Bessede et al. nicely demonstrated that TDO^−/−^ mice exhibit an increased sensitivity to endotoxin-induced shock, indicating the potential relevance of TDO in anti-inflammatory reactions [5]. Further hints towards an immunoregulatory function of TDO derive from the facts that TDO is expressed in hepatocarcinomas and other malignancies and TDO-mediated production of tryptophan metabolites protects tumor cells against immune rejection [6–9]. Interestingly, hypoxia is frequently observed in tumoral tissue and influences host defence [10–12]. Hence, hypoxia is a microenvironmental factor that might have an impact also on TDO-mediated functions.

It was shown by us that recombinantly expressed human TDO is able to inhibit the growth of bacteria, parasites, and viruses [4] and also infected tissue displays low oxygen levels or hypoxia [12]. Several factors contribute to these infection- or inflammation-associated hypoxic states, for example, increased oxygen consumption by inflamed resident cells, infiltrating inflammatory cells, and proliferating pathogens as well as a decreased oxygen supply due to vascular pathology and microthrombosis [13].

Here we were interested in TDO functions under hypoxia. Using stably transfected HeLa T-REx*™* cells expressing recombinant human TDO [4] and liver homogenates from WT and TDO^−/−^ mice we found that the TDO-mediated degradation of tryptophan to kynurenine was inhibited under low oxygen concentrations. Consequently the antimicrobial functions of TDO against tryptophan-auxotroph bacteria and parasites were abrogated* in vitro*. In summary our studies revealed that low oxygen levels might be detrimental for antimicrobial effector molecules (IDO, TDO), inhibiting appropriate immune reactions during infections, which possibly leads to inadequate microbial clearance and subsequent overwhelming or chronic infections.

## 2. Methods

### 2.1. Cells, Media, and Reagents

HeLa T-REx cells were purchased from Invitrogen (Karlsruhe, Germany) and stably transfected with pcDNA4-hTDO vector (Invitrogen, Karlsruhe, Germany) containing human liver TDO cDNA to produce HeLa-hTDO cells as described by us [4]. The expression of recombinant hTDO in HeLa-hTDO cells was induced by stimulation with tetracycline.

The cells were cultured in Iscove's modified Dulbecco's medium (IMDM) (Gibco, Grand Island, USA), supplied with 5% heat-inactivated fetal calf serum (FCS) in culture flasks (Costar, Cambridge, USA) and split weekly in 1 : 10 ratios by using trypsin/EDTA (Gibco, Grand Island, USA). Mycoplasma contamination was regularly excluded via PCR. Hypoxia growth experiments were carried out using alternatively a HERAcell 150 I CO_2_ incubator (Thermo Fisher Scientific, Langenselbold, Germany) or the Anoxomat*™* system (Mart Microbiology B.V., Drachten, Netherlands) with 1–10% O_2_ and 10% CO_2_. The IMDM was buffered with sodium bicarbonate and therefore had an optimal buffering capacity at the 10% CO_2_ environment to maintain the physiological pH. Consequently the pH values of the cell culture medium with or without cells under normoxia and hypoxia were in the range of 7.15–7.48.

### 2.2. Cell Proliferation Assay

1 × 10^3^ HeLa-hTDO cells were seeded per cm^2^ in 25 cm^2^ cell culture flask (Corning, NY, USA), ^3^H-thymidine was added at day 0, and the cells were incubated under normoxia (20% O_2_) or hypoxia (1% O_2_) for 1–7 days. The incorporation of ^3^H-thymidine was detected using liquid scintillation spectrometry (1205 Betaplate, PerkinElmer, Rodgau Jügesheim, Germany).

### 2.3. Cell Viability Assay

3 × 10^4^ HeLa-hTDO cells per well were incubated in 96-well Costar microtiter plates (Corning, NY, USA) for 24 h under normoxia (20% O_2_) or hypoxia (1% O_2_). Then the cells were washed with PBS and stained with Calcein AM (1 : 2000) or Ethidium-Homodimer-1 (Eth-D-1) (1 : 500) (live/dead viability/cytotoxicity kit, Invitrogen, Karlsruhe, Germany). After incubation time of 45 minutes the fluorescence was detected using the plate reader Synergy Mx (Winooski, VT, USA). Calcein was excited using a fluorescein optical filter (485 ± 10 nm) and Eth-D-1 using a rhodamine optical filter (530 ± 12 nm). The fluorescence emissions were acquired separately as well, Calcein at 530 ± 12 nm and Eth-D-1 at 645 ± 12 nm.

### 2.4. Enzyme Activity Assays

#### 2.4.1. Detection of Kynurenine in Cell Supernatants

TDO activity was determined by analysing kynurenine concentration in supernatants of stimulated or unstimulated HeLa-hTDO, using Ehrlich's reagent as described before [14]. Defined kynurenine samples served as control.

#### 2.4.2. Assessment of mTDO Activity in Liver Homogenates

Assessment of mTDO activity in liver homogenates was done according to the method of Moreau et al. [15], with some modifications: livers were homogenized in three times the weight using a tissue homogenizer (Precellys, VWR International, Erlangen, Germany). 400 *μ*L liver homogenisate was added to 1 mL TDO-buffer (200 mM potassium phosphate buffer (pH 7.0), 10 mM ascorbic acid, 5 *μ*M hematin, and 10 mM L-tryptophan) in 48-well microtiter plates and was incubated without lid under normoxia (20% O_2_) or hypoxia (1% O_2_) for 4 h at 37°C. Then the reaction was stopped using 1/10 v/v 30% trichloroacetic acid, again incubated for 30 min at 60°C, and centrifuged. Supernatants were mixed with an equal volume of Ehrlich's reagent and absorbance was detected at 492 nm with a microplate reader (SLT Lab Instruments, Crailsheim, Germany).

Kynurenine (Sigma-Aldrich, St. Louis, USA) was diluted in culture medium or buffer and used as standard. For the calculation of the kynurenine content, linear regression and GraphPad Prism software were used.

### 2.5. *In Vitro* Infection Experiments

For* in vitro* infection studies 3 × 10^4^ HeLa-hTDO were incubated under normoxia (20% O_2_) or hypoxia (1% O_2_) in the presence or absence of tetracycline for 72 h. Thereafter they were infected with different bacteria or parasites.

#### 2.5.1. Bacterial Infections and Read-Out

For bacterial infections* Enterococcus faecalis* (ATCC 29212) was used. Tryptophan auxotrophy was tested before starting infection experiments. Bacteria were grown on brain heart infusion agar (Difco, Hamburg, Germany), containing 5% sheep blood and incubated at 37°C in 5% CO_2_-enriched atmosphere or in cell culture medium in the absence or presence of cells. Tetracycline-sensitivity was tested and a half-maximal bacterial growth was observed in the presence of 40 *μ*g/mL tetracycline under normoxia and under hypoxia, which is thousandfold more than the concentration we used in the experiments. For use in experiments, a 24 h old single bacterial colony was picked, resuspended in tryptophan-free RPMI 1640 (Gibco, Life Technologies, Darmstadt, Germany), and diluted serially. Ten *μ*L of the bacterial suspension corresponding to 10–100 CFU was added to each well of the 96-well microtiter plates containing preincubated HeLa-hTDO cells. After 18 h bacterial growth was monitored using a microplate photometer (SLT Lab Instruments, Crailsheim, Germany) by measuring the optical density at 620 nm. In some experiments, the bacterial population present in the cultures was enumerated by counting colony forming units (CFU) after plating 10 *μ*L aliquots of serially diluted culture supernatants on blood agar [16].

#### 2.5.2. Parasite Infections and Read-Out

*Toxoplasma gondii* (RH strain, ATCC, Wesel, Germany) or* Neospora caninum* (Nc-1 strain, kind gift of G. Schares, Greifswald-Insel Riems, Germany) tachyzoites were maintained in human foreskin fibroblasts (ATCC, Wesel, Germany) in IMDM containing 5% FCS. Tachyzoites were harvested after 5 days of incubation, resuspended in PBS, and counted. Preincubated HeLa-hTDO cells were infected with 3 × 10^4^ toxoplasma or 4 × 10^4^ neospora per well. Parasite growth was determined by the ^3^H-uracil incorporation method as described before [17]. In brief 48 h after infection 0.33 *μ*Ci ^3^H-uracil was added and after additional 24 h host cells were lysed by freeze and thaw cycles. The incorporation of ^3^H-uracil was detected using liquid scintillation spectrometry (1205 Betaplate, PerkinElmer, Rodgau Jügesheim, Germany).

### 2.6. Protein Analysis

3 × 10^6^ HeLa-TDO cells were left unstimulated or stimulated with tetracycline (10 ng/mL) for 72 h under normoxia (20% O_2_) or hypoxia (1% O_2_). Then the cells were harvested and lysed by three freeze and thaw cycles in a protease inhibitor cocktail (Roche Diagnostics GmbH, Mannheim, Germany). Proteins were separated by electrophoresis using 10% NuPAGE Novex* Bis*-Tris Mini Gels in the appropriate electrophoresis system (Invitrogen, Karlsruhe, Germany) and semidry blotted on nitrocellulose membranes (CarboGlas, Schleicher & Schuell, Dassel, Germany). After blocking of the membranes with 3% (w/v) skim milk powder in TBS for 1 h at room temperature, they were incubated in the respective primary antibodies overnight at 4°C. Anti-*β*-actin antibody (1 : 10000, Sigma, St. Louis, USA) or anti-human-TDO2 antibody (GTX 40401, GeneTex, Irvine, USA) was diluted in 3% (w/v) skim milk powder in TBS. After washing the membranes were incubated with goat-anti-mouse HRP-conjugated or goat-anti-rabbit HRP-conjugated IgG (1 : 10000, Jackson ImmunoResearch Lab., Dianova, Hamburg, Germany), diluted in 3% (w/v) skim milk powder in TBS, for 2 h at room temperature. After additional washing steps proteins were detected by enhanced chemiluminescence (Amersham Pharmacia Biotech, Freiburg, Germany). Densitometric analysis was carried out with ImageJ software.

### 2.7. T Cell Proliferation Assay

3 × 10^6^ HeLa-hTDO cells were incubated with or without tetracycline (10 ng/mL) for 72 h under normoxia (20% O_2_) or hypoxia (1% O_2_) in 20 mL cell culture medium in culture flasks. Then the supernatants were harvested and used as cell culture medium for freshly isolated 1.5 × 10^5^ Ficoll-separated peripheral blood lymphocytes (PBL)/well. PBL were activated using the monoclonal anti-CD3 antibody OKT3; unstimulated PBL and tryptophan-supplemented PBL served as control group. T cell proliferation was determined after three days by adding ^3^H-thymidine for 24 h. The incorporation of ^3^H-thymidine was detected using liquid scintillation spectrometry (1205 Betaplate, PerkinElmer, Rodgau Jügesheim, Germany).

### 2.8. Animals

This study was carried out in strict accordance with the German Animal Welfare Act and a protocol approved by the local authorities. TDO-deficient mice were generated as described previously [18]. WT littermates were used as controls. Mice were housed under SPF conditions in the animal facility and were 8–12 weeks old.

### 2.9. Data Analysis and Statistical Tests

**Figure 1:**
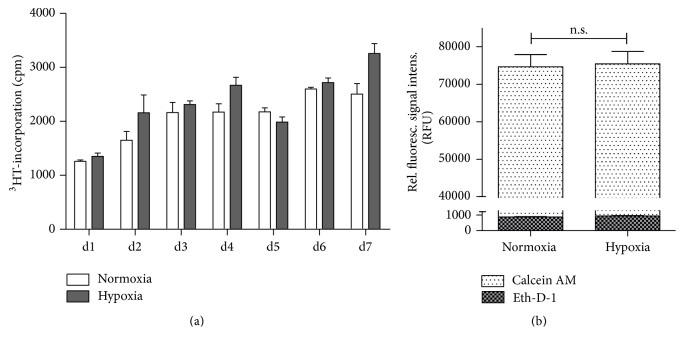
HeLa-hTDO cells survive incubation under hypoxic conditions. (a) Proliferation assay: 1 × 10^3^ HeLa-hTDO cells were seeded per cm^2^ in 25 cm^2^ cell culture flask (Corning, NY, USA), ^3^H-thymidine was added at day 0, and the cells were incubated under normoxia (20% O_2_) or hypoxia (1% O_2_) for 1–7 days. The incorporation of ^3^H-thymidine was detected using liquid scintillation spectrometry (1205 Betaplate, PerkinElmer, Rodgau Jügesheim, Germany). (b) Fluorescence-based cell viability/cytotoxicity assays: 3 × 10^4^ HeLa-hTDO cells per well were incubated for 24 h under normoxia (20% O_2_) or hypoxia (1% O_2_). Then the cells were stained with Calcein AM (white bars) or Ethidium-Homodimer-1 (Eth-D-1) (plaid bars) indicating living or dead cells, respectively. After incubation the fluorescence was detected using a fluorescein optical filter (excitation 485 ± 10 nm; emission 530 ± 12 nm) for Calcein or using a rhodamine optical filter (excitation 530 ± 12 nm; emission 645 ± 12 nm) to detect Eth-D-1. For both assays a two-tailed unpaired* t*-test was used to compare the groups (n.s. = not significant), *n* = 3 independent experiments with three replicates each. The bars indicate the mean value ± SEM.

**Figure 2 fig2:**
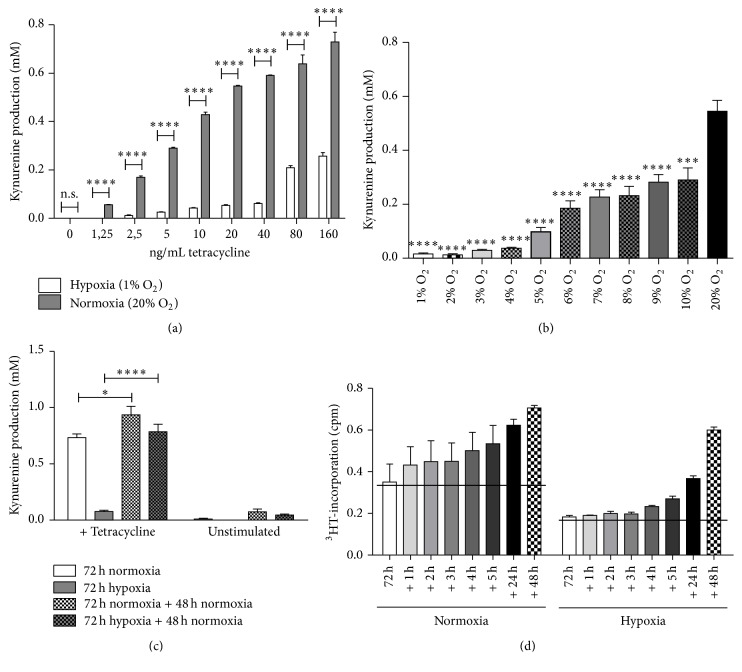
Loss of TDO activity in HeLa-hTDO cells under hypoxic conditions. (a) Kynurenine detection in cell culture supernatants of tetracycline (0–160 ng/mL) stimulated HeLa-hTDO cells incubated under hypoxia (white bars) or normoxia (grey bars). (b) Kynurenine detection in cell culture supernatants in tetracycline stimulated (160 ng/mL) HeLa-hTDO cells that have been incubated under different oxygen conditions (1–20% O_2_) for 72 h. (c + d) Reoxygenation study: HeLa-hTDO cells were incubated under normoxia (20% O_2_) or hypoxia (1% O_2_) with or without tetracycline (40 ng/mL) for 72 h. Then the kynurenine amount produced by TDO was detected in cell culture supernatants. A second experimental group was subsequently transferred to normoxia and after incubation of additional 48 h (c) or 1–5 h, 24 h, and 48 h (d) the kynurenine amount was also detected in cell culture supernatants. In all experiments a significant alteration of kynurenine production under hypoxia as compared to the normoxia control is marked with asterisks (^*∗*^ *p* ≤ 0.05; ^*∗∗∗*^ *p* ≤ 0.001; ^*∗∗∗∗*^ *p* ≤ 0.0001, n.s. = not significant), two-tailed unpaired* t*-test; *n* = 3 independent experiments with three replicates each. The bars indicate the mean value ± SEM.

**Figure 3 fig3:**
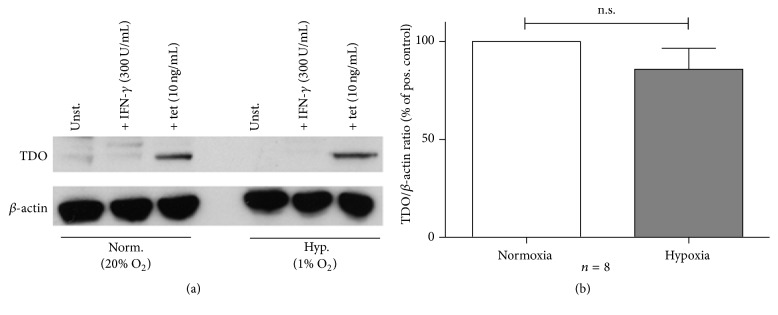
Unaltered hTDO expression in HeLa-hTDO cells under hypoxic conditions. (a) Exemplary Western Blot protein analysis of hTDO and *β*-actin protein expression in HeLa-hTDO cells after 72 h of incubation under normoxia (20% O_2_) or hypoxia (1% O_2_). (b) Densitometric evaluation of Western Blot protein analyses: ratio of relative hTDO protein expression to *β*-actin protein expression as % of positive control ± SEM, *n* = 8 independent experiments. Comparison of hTDO/*β*-actin protein ratio under normoxia or hypoxia via a two-tailed, paired* t*-test; n.s. = not significant.

**Figure 4:**
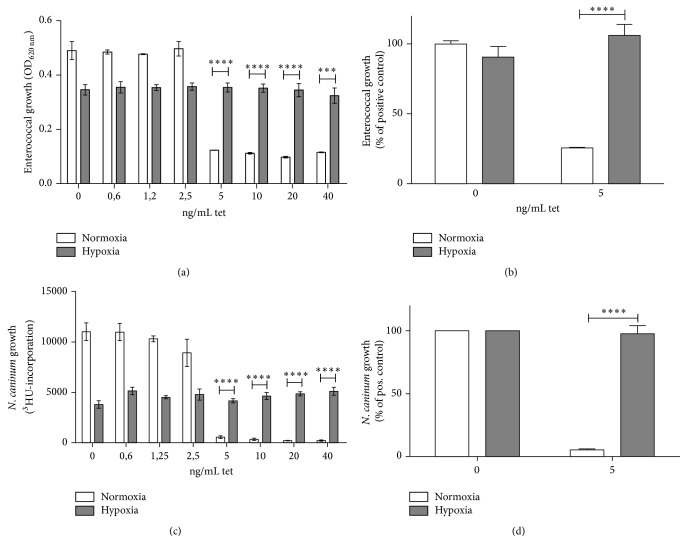
Loss of TDO-mediated antimicrobial functions in HeLa-hTDO cells under hypoxic conditions. Infection experiments: following 72 h prestimulation with denoted amounts of tetracycline, HeLa-hTDO cells infected with* Enterococcus faecalis* (a and b) or* Neospora caninum* (Nc-1 strain) (c and d). After 24 h the bacterial growth or after 48 h the parasite growth was determined by measurement of OD_620 nm_ or by the ^3^H-uracil incorporation method, respectively. (a and c) Single experiment with three replicates and (b and d) summary of three independent experiments with three replicates each. A significant decrease of microbial growth under hypoxia as compared to the normoxia control is marked with asterisks (^*∗∗∗*^ *p* ≤ 0.001; ^*∗∗∗∗*^ *p* ≤ 0.0001) and was calculated by a two-tailed, unpaired* t*-test. The bars indicate the mean value ± SD (a and c) or ± SEM (b and d).

**Figure 5:**
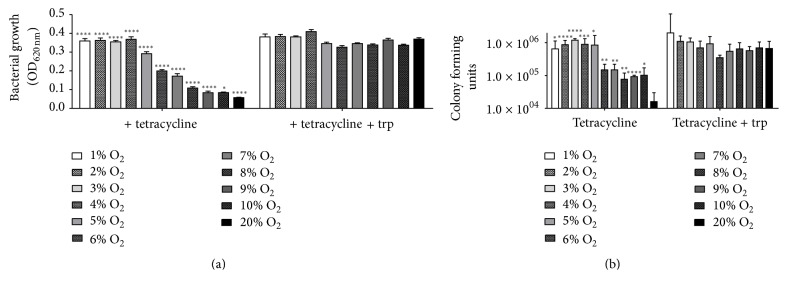
Loss of TDO-mediated antibacterial functions in HeLa-hTDO cells under low oxygen conditions. Infection experiments: HeLa-hTDO cells were incubated under different oxygen conditions (1–20% O_2_) for 72 h with or without tetracycline (160 ng/mL) and afterwards infected with* Enterococcus faecalis* in the presence or absence of additional tryptophan. (a) After 24 h the bacterial growth was determined by measurement of OD_620 nm_, in 3 independent experiments with three replicates each. (b) Determination of colony forming units by serial dilution and summary of 7 independent experiments. Significant alterations of microbial growth as compared to the normoxia control are marked with asterisks (^*∗*^ *p* ≤ 0.05; ^*∗∗*^ *p* ≤ 0.01; ^*∗∗∗*^ *p* ≤ 0.001; ^*∗∗∗∗*^ *p* ≤ 0.0001) and were calculated by a two-tailed, unpaired* t*-test. The bars indicate the mean value ± SEM (a) or SD (b).

**Figure 6:**
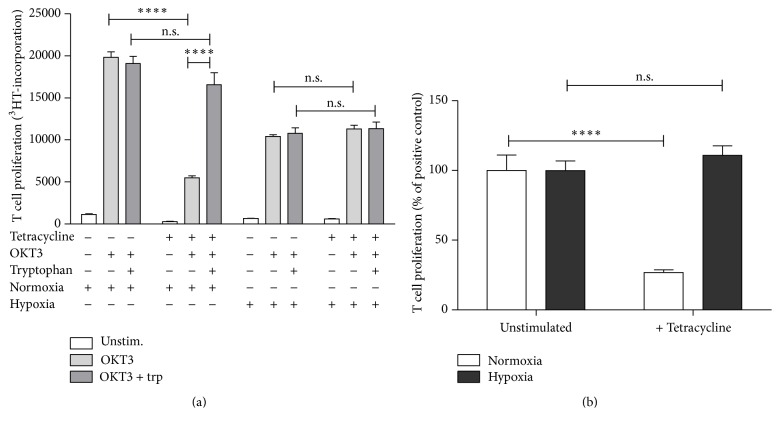
Loss of TDO immunoregulatory function in HeLa-hTDO cells under hypoxic conditions. T cell proliferation experiments: HeLa-hTDO cells were prestimulated with or without tetracycline (10 ng/mL) for 72 h under normoxia (20% O_2_) or hypoxia (1% O_2_). Then the supernatants were harvested and served as cell culture medium for freshly isolated peripheral blood lymphocytes (PBL). 1.5 × 10^5^ PBL/well were activated in 96-well plates with a monoclonal anti-CD3 antibody (OKT3) and T cell proliferation was determined after three days by adding ^3^H-thymidine for 24 h. The incorporation of ^3^H-thymidine was detected using liquid scintillation spectrometry (1205 Betaplate, PerkinElmer, Rodgau Jügesheim, Germany). A significant alteration of T cell proliferation as compared to the respective control group is marked with asterisks (^*∗∗∗∗*^ *p* ≤ 0.0001; n.s. = not significant) and was calculated via a two-tailed, unpaired* t*-test. (a) Single experiment with three replicates and (b) summary of three independent experiments with three replicates each. The bars indicate the mean value ± SD (a) or ± SEM (b).

**Figure 7:**
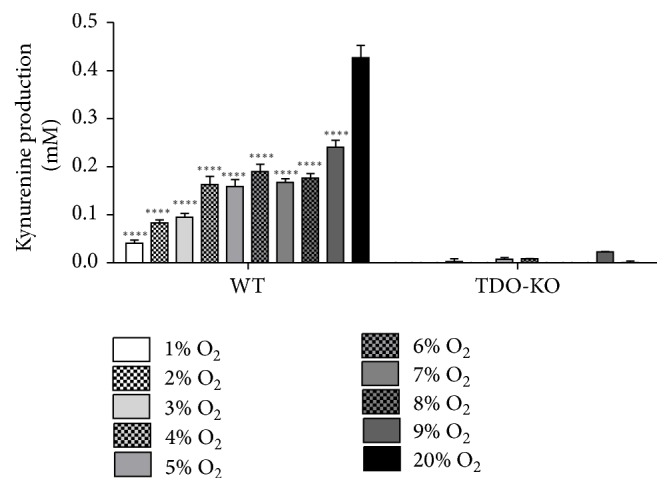
Loss of TDO activity in liver homogenates under hypoxic conditions. Determination of murine TDO* ex vivo* activity. Livers of WT or TDO-deficient C57BL/6 mice were homogenized in PBS and liver homogenisates were added to TDO-buffer in 48-well microtiter plates, followed by an incubation under normoxia (20% O_2_) or other oxygen amounts (1–9% O_2_) for 4 h at 37°C. Then the reaction was stopped using 30% trichloroacetic acid and the kynurenine amount was determined in the supernatants via supplementation of Ehrlich's reagent and absorbance at 492 nm. *n* = 3 independent experiments with three replicates each. A significant alteration of kynurenine levels as compared to the normoxia control is marked with asterisks (^*∗∗∗∗*^ *p* ≤ 0.0001), as determined via a two-tailed, unpaired* t*-test. The bars indicate the mean value ± SEM.

All experiments were done in triplicate and data are given as mean ± standard deviation (SD; Figures 1(a) [Fig fig2] [Fig fig3], 4(a), 4(c), 5(a), and 6(a)) of a representative experiment or as mean ± standard error of the mean (SEM; Figures 1(b), 2(a)–2(c), 3, 4(b), 4(d), 5(b), 6(b), and 7) of three to eight independent experiments. For statistical analysis the two-tailed paired* t*-test (Figure 3) or the two-tailed unpaired* t*-test (all other data) was used and significant differences were marked with asterisks (^*∗*^ *p* ≤ 0.05; ^*∗∗*^ *p* ≤ 0.01; ^*∗∗∗*^ *p* ≤ 0.001; ^*∗∗∗∗*^ *p* ≤ 0.0001). The analysis was performed with GraphPad Prism software (GraphPad Software Inc., San Diego, CA).

## 3. Results

### 3.1. HeLa-hTDO Survives Incubation under Hypoxia

We have shown before that the tryptophan-degrading enzyme human tryptophan 2,3-dioxygenase (hTDO), expressed in a tetracycline-inducible HeLa-cell based system, has antibacterial, antiparasitic, and antiviral capacities* in vitro* [4]. These antimicrobial effects are the result of the hTDO-induced degradation of tryptophan, which is an essential amino acid for tryptophan-auxotroph organisms, since the supplementation with additional tryptophan abrogated the effects.

In order to analyse hTDO-mediated antimicrobial effects under hypoxic conditions, we first monitored HeLa-hTDO cell survival under hypoxia. Proliferation studies revealed no significant differences in cell growth, when HeLa-hTDO cells were cultured under normoxia or hypoxia (1% O_2_) for 7 days (Figure 1(a)). Additionally we observed no significant alterations in cell proliferation under normoxia or hypoxia when the cells were stimulated with tetracycline (data not shown). Therefore we concluded that the HeLa-hTDO cells thoroughly survive the hypoxic environment, at least beyond the three-day incubation phases in the subsequent analyses. This cell survival was also confirmed using a fluorescence-based method of assessing cell viability and cell death (Figure 1(b)). For these experiments HeLa-hTDO cells were incubated for 24 h under normoxia (20% O_2_) or hypoxia (1% O_2_) and afterwards stained with Calcein AM to detect intracellular esterase activity as indication for living cells or Ethidium-Homodimer-1 (Eth-D-1) that enters nonintact plasma membranes of dead cells. Figure 1(b) shows that the relative fluorescence signal intensity after staining with Calcein AM does not significantly differ in normoxia- or hypoxia-treated HeLa-hTDO cells (white bars). Furthermore Eth-D-1 treatment revealed no enhanced cell death under hypoxia (plaid bars).

### 3.2. Enzymatic Activity of Human TDO Is Reduced upon Hypoxia

The enzymatic activity of hTDO under hypoxia was analysed by determination of the tetracycline-induced hTDO-mediated conversion of tryptophan to kynurenine in cell culture supernatants after 72 h of incubation. Tetracycline stimulated HeLa-hTDO cells produced kynurenine dose-dependently, when they were incubated under normoxia in the presence of tryptophan (Figure 2(a)). Interestingly the cells generated significantly lower amounts of kynurenine under hypoxic conditions (1% O_2_) (Figure 2(a)). When tetracycline stimulated cells were incubated under different oxygen concentrations, the kynurenine amounts positively correlated with the amounts of oxygen present (Figure 2(b)). Therefore oxygen was required for the production of kynurenine. Reoxygenation studies confirmed survival of HeLa-hTDO cells and preservation of enzymatic function within these cells under hypoxic conditions (Figure 2(c)). In these experiments the tetracycline-induced TDO-mediated kynurenine production was determined after 72 h of incubation under normoxia (20% O_2_) or hypoxia (1% O_2_) and compared to the kynurenine production within cell supernatants after subsequent 48 h incubation under normoxia. The TDO-mediated conversion of tryptophan to kynurenine was drastically inhibited by hypoxia, but the cells were able to produce high amounts of kynurenine in the following normoxic phase, demonstrating cell survival and preservation of enzymatic activity. Interestingly, normoxia-pretreated and hypoxia-pretreated cells showed no significant differences in their tryptophan-degrading capacity in the reoxygenation phase* ab initio* (Figure 2(d)).

### 3.3. Expression of Human TDO under Hypoxic Conditions

Next, Western Blot analyses were performed to get quantitative information about hTDO protein amounts in HeLa-hTDO cells that were stimulated under normoxia (20% O_2_) and hypoxia (1% O_2_).  Figure 3(a) depicts an exemplary Western Blot. The protein amount of hTDO, induced by tetracycline stimulation of HeLa-hTDO cells, was not altered upon hypoxia as compared to the normoxia control. The stimulation of the cells with the proinflammatory cytokine IFN-*γ* did not induce a TDO expression in the cells, as expected. The summary of eight densitometric evaluations of independent Western Blot analyses is shown in Figure 3(b). Since *β*-actin protein amounts are also reduced upon hypoxia [19], the ratio of hTDO protein to *β*-actin protein was calculated. There were no significant differences detectable in hTDO protein amounts under normoxic and hypoxic conditions detectable.

### 3.4. Inhibition of hTDO-Mediated Antimicrobial Effects by Hypoxia

Although hTDO protein levels were unaltered under hypoxia (Figure 3), the enzymatic activity of hTDO was reduced up to nearly 90%, as determined by measurement of kynurenine in HeLa-hTDO cell supernatants after 72 h of incubation under normoxia (20% O_2_) or hypoxia (1% O_2_) (Figure 2). In order to analyse hTDO-mediated antimicrobial functions under hypoxia, we infected unstimulated or tetracycline prestimulated HeLa-hTDO cells with different tryptophan-auxotroph pathogens.  Figure 4(a) shows the result of infection experiments with the facultative anaerobe, gram-positive bacterium* Enterococcus faecalis.* Enterococci grew in the presence of unstimulated HeLa-hTDO cells, whereas bacterial growth was inhibited by TDO-positive cells, which have been stimulated with ≥5 ng/mL tetracycline (white bars) under normoxic conditions. Interestingly, this TDO-mediated antibacterial effect was lost under hypoxic conditions (grey bars). The same result was observed in infection experiments using other tryptophan-auxotroph bacteria, such as group B streptococci and staphylococci (data not shown).  Figure 4(b) shows that moreover the growth of the obligate intracellular apicomplexan parasite* Neospora caninum* (nc-1 strain) was inhibited within activated HeLa-hTDO cells under normoxia and that this antiparasitic effect was abolished under hypoxic conditions.

A more detailed analysis of the hTDO-mediated antibacterial effect under different low oxygen conditions (1–10% O_2_) in comparison to normoxia is shown in Figure 5. The antibacterial efficiency of hTDO correlated with the presence of oxygen and low oxygen conditions significantly inhibited the antibacterial effect as determined by the optical density (Figure 5(a)) or by counting the colony forming units (Figure 5(b)).

### 3.5. Inhibition of hTDO-Mediated Immunoregulatory Effects by Hypoxia

We have mentioned before that TDO regulates immune reactions* in vitro* and* in vivo*, for example, by creating tolerance towards the rejection of tumor cells [6]. Furthermore tumoral tissues are often poorly vascularized and inefficiently supplied with blood and contain only low oxygen amounts [10, 11]. Hence, we tested the immunoregulatory property of hTDO in HeLa-hTDO cells under hypoxia* in vitro*. In order to avoid IFN-*γ* production by allogeneic T cells which would result in IDO induction in HeLa cells by coculture, supernatants of tetracycline-activated or unstimulated and hypoxia- or normoxia-treated HeLa-hTDO served as culture medium for freshly isolated peripheral blood lymphocytes (PBL). T cell growth was triggered by the addition of the monoclonal anti-CD3 antibody OKT3 and monitored by the ^3^H-thymidine incorporation method [4]. This OKT3 stimulation induced strong T cell proliferation, which is illustrated in a single experiment in Figure 6(a). The T cell proliferation was inhibited by the TDO-mediated depletion of tryptophan, since it could be restored by the supplementation of tryptophan. However, such T cell inhibition did not occur in conditioned medium that has been harvested from hypoxia-treated HeLa-hTDO cells, which demonstrates the loss of immunoregulatory hTDO functions under hypoxia.  Figure 6(b) shows the summary obtained from three different experiments.

### 3.6. *Ex Vivo* TDO Activity under Hypoxic Conditions

Although stably transfected HeLa cells are a useful tool to efficiently examine hTDO functions* in vitro*, we extended our studies and confirmed the data by more physiological* ex vivo* liver homogenate experiments. In these experiments freshly isolated liver tissue of WT or TDO-deficient mice was homogenized in PBS and incubated in the presence of 20% O_2_ or 1–9% O_2_ in a buffer that allows the determination of TDO protein activity by the production of kynurenine [15]. Since the normoxic and the hypoxic groups contained the same amounts of murine TDO protein, the direct effect of normoxia and hypoxia on TDO enzyme activity could be revealed (Figure 7). Under hypoxia murine TDO produced significantly lower kynurenine amounts as compared to the normoxia control group. Furthermore, no kynurenine was detectable in liver homogenates of TDO-deficient mice, as expected.

## 4. Discussion 

In this study we investigated the influence of hypoxia on the activity of the tryptophan-degrading enzyme human tryptophan 2,3-dioxygenase (hTDO) by using a the tetracycline-inducible HeLa T-REx system together with* ex vivo* studies analysing murine TDO [4]. Under normoxic conditions (20% O_2_) hTDO activity reduced tryptophan amounts in tetracycline stimulated HeLa-hTDO cells and cell culture supernatants [4]. However, such high oxygen concentrations do not occur within liver tissue physiologically. The liver has a unique anatomical structure that creates an oxygen gradient within the liver compartments. Incoming highly oxygenated blood via the hepatic artery is subsequently mixed with oxygen-depleted blood in the hepatic portal vein. Then the blood flows towards the central vein of the lobule and is depleted of oxygen, resulting in an oxygen pressure (pO_2_) of about 8% O_2_ in the periportal area and of about 4% O_2_ in parenchymatic perivenous areas [2]. Additionally, oxygen concentrations in the liver are even more reduced upon infection. For example, pO_2_ of ~1.3% was detected within the liver tissue of* Schistosoma mansoni*-infected mice [3]. Furthermore a high expression rate of the hypoxia inducible factor-1*α* (HIF-1*α*) was detected in the infiltrative belt surrounding hepatic alveolar echinococcosis lesions within rat livers [20]. Overexpression of HIF-1*α* in the actively multiplying infiltrative region of these lesions was closely related to angiogenesis and microvasculature [20]. Similar pO_2_ levels of approximately 1 to 3% O_2_ were detected in other inflamed and infected tissues in the skin, the lungs, and the gut [12]. For example,* Leishmania major* infected mice display low oxygen levels of about 2.8% O_2_ in pronounced skin lesions, while the resolution of the wound was accompanied by an increase of lesional oxygen levels [21]. Hence there is a correlation between infected and inflamed tissues and low oxygen amounts within these tissues. Given the fact that hTDO has antimicrobial properties under normoxic conditions* in vitro* and that a microbial-induced hypoxic state is detected within liver tissue, we checked whether putative hTDO-mediated antimicrobial effects might persist under low oxygen conditions. Therefore the expression and activity of recombinant human TDO in transfected HeLa cells as well as* ex vivo* murine TDO were analysed under normoxic and hypoxic conditions. In first step the survival of HeLa-hTDO cells within a hypoxic microenvironment of 1% O_2_ was confirmed in cell proliferation tests, in fluorescence-based cell viability/cytotoxicity assays and in reoxygenation-based enzyme activity studies. All of these tests provided no indication for an enhanced hypoxia-induced cell death of HeLa-hTDO cells. Then the enzymatic activity of hTDO was determined by measurement of kynurenine, which is the product of the TDO-mediated tryptophan degradation. Human TDO efficiently catalysed the formation of kynurenine under normoxic conditions (20% O_2_), whereas significantly lower levels of kynurenine were generated under oxygen concentrations detected in liver tissue physiologically (1–10% O_2_). Our* ex vivo* studies using liver homogenates from wildtype and TDO-deficient mice clearly show that also the mTDO-dependent degradation of tryptophan is significantly reduced under low oxygen conditions (<9% O_2_). In particular the low pO_2_ of 1–3% O_2_, which has been detected in infected liver tissue significantly restricted the enzymatic activity of human and murine TDO enzymes. The reason for the lower tryptophan conversion rate under hypoxic conditions* in vitro* could be general downregulation of TDO protein levels or a reduced enzymatic function. The analysis of human TDO protein amounts within normoxia- and hypoxia-treated HeLa-hTDO cells via Western Blot analyses showed no decrease of hTDO protein amounts under hypoxia. HTDO protein levels were correlated with the respective *β*-actin band, since it is known that hypoxia caused general alterations in protein expression levels [18]. Therefore a decrease in hTDO expression could not account for the decrease of hTDO activity under hypoxia. Hence the enzymatic activity of hTDO must be altered, matching the fact that TDO is a protoheme-containing enzyme that catalyses the insertion of O_2_ into the pyrrole ring of L-tryptophan and is therefore dependent on cellular oxygen portions [22]. In line with that we observed that normoxia-pretreated and hypoxia-pretreated cells showed no significant differences in their tryptophan-degrading capacity in the reoxygenation phase* ab initio.*

A second dioxygenase that likewise catalyses the degradation of tryptophan is the enzyme indoleamine 2,3-dioxygenase (IDO). Already in the 1980s it was shown that the intracellular degradation of tryptophan, induced by IFN-*γ*, restricted the growth of the intracellular parasite* Toxoplasma gondii* in human fibroblasts [23]. Since then IDO crystallized as broad-spectrum antimicrobial effector molecule that is effective against a variety of tryptophan-auxotroph pathogens* in vitro* [24]. Interestingly, IDO-mediated tryptophan depletion is also inhibited under low oxygen concentrations (≤3% O_2_) which results in a loss of IDO-mediated antimicrobial effects* in vitro* [19, 25, 26]. This was first shown in infection experiments using the intracellular bacterium* Chlamydia trachomatis* in human fallopian tube cells and pinpointed to the hypoxia-dependent inhibition of IFN-*γ* signalling [26]. This loss of IDO-mediated antimicrobial effects under hypoxia raised the question to what extent TDO might lose its antimicrobial properties upon hypoxic circumstances.

Here we show that the antibacterial and antiparasitic effects of hTDO were lost in HeLa-hTDO cells under hypoxia. Human TDO was no longer able to inhibit the growth of* Enterococcus faecalis* and other tryptophan-auxotroph bacteria such as* Staphylococcus aureus* or group B streptococci (data not shown). Furthermore the hTDO-mediated defence against the intracellular parasite* Neospora caninum* was lost under hypoxic conditions. Since hTDO is expressed within HeLa-hTDO cells by the addition of tetracycline and not by IFN-*γ* stimulation, perturbations in the IFN-*γ* signalling pathway cannot account for these observations, but only the lack of molecular oxygen.

Unfortunately it is impossible to analyse potential antimicrobial functions of mTDO or hTDO in isolated primary hepatocytes, since these cells readily undergo dedifferentiation and lose hepatocyte function [27]. Therefore further studies need to be performed analysing such TDO functions in* ex vivo* induced stem cells (e.g., embryonic stem cells, pluripotent stem cells, and hepatic progenitor cells) that are differentiated into hepatocyte-like cells with potential TDO function.

Since there are also no data claiming for an antimicrobial function of mTDO* in vivo* up to now, the immunoregulatory function of TDO is in the focus of research. Here we show that also the T cell inhibitory function of hTDO was lost in HeLa-hTDO cells upon hypoxic conditions. Again hypoxia prevented hTDO-mediated tryptophan depletion and kynurenine production which led to unhindered OKT3-driven T cell proliferation in supernatants of hTDO-positive HeLa-hTDO cells. In consistence with findings from other groups we detected reduced overall T cell proliferation under hypoxia. For example, Atkuri et al. clearly demonstrated that the influence of oxygen levels on the T cell proliferation depends on the stimulus used to activate T cells. While the proliferation in response to phytohemagglutinin was not altered under different oxygen conditions, the CD3/CD28 crosslinking and the stimulation with Con A lead to significant higher proliferation under atmospheric oxygen levels than under physiologic oxygen levels (5% and 10% O_2_), with the latter being comparable to our experimental setting [28]. This observation might be of relevance* in vivo*, since hypoxia is frequently observed in tumoural tissue [10, 11]. Tumor cells, as well as healthy cells, adapt to hypoxia by various appropriate physiologic responses, for example, by altered expression of genes that switch from oxidative to glycolytic metabolism [29, 30]. These cellular responses are caused by the induction of the hypoxia-inducible factor (HIF) protein complex due to hypoxia. The HIF complex regulates the expression of more than 100 genes included in metabolism, angiogenesis, vascular tone, cell differentiation, and apoptosis, among them various enzymes [31]. TDO mRNA is frequently expressed within human hepatocarcinomas, but information about TDO activity is missing and the cellular source is still unknown [6, 7]. TDO-mediated production of tryptophan metabolites protects TDO-transfected tumour cells against immune rejection [8, 9]. Therefore the role of TDO in immunoregulation is crucial.

Our data indicate hypoxia as an environmental factor which is present in tumoural tissue that strongly impacts TDO activities and might therefore be beneficial for tumour growth.

## 5. Conclusions

The strong influence of low oxygen amounts on innate and adaptive immunity in both inflamed resident cells and infiltrating immune cells was described before [32–34]. Since several antimicrobial and immunoregulatory effector molecules in addition to TDO and IDO, as, for example, the phagocyte NADPH oxidase (PHOX), type 2 nitric oxide synthase (NOS2), and mitochondria rely on molecular oxygen as a substrate, hypoxia impairs their activity, which might promote the survival of pathogens [12]. Therefore low oxygen levels might lead to an inadequate control of microorganisms and to subsequent overwhelming or chronic infections. The role of TDO in this context has still to be identified.
